# Investigating the effect strength of positive risk-taking barriers on discharge decisions in occupational therapy intermediate care: A factorial survey

**DOI:** 10.1177/03080226221141320

**Published:** 2022-12-13

**Authors:** Craig Newman, Phillip Whitehead, Mary Thomson

**Affiliations:** 1 Department of Social Work, Education & Community Wellbeing, Northumbria University, Newcastle upon Tyne, UK; 2 Population Health Sciences Institute, Faculty of Medical Sciences, Newcastle University, Newcastle upon Tyne, UK; 3 Newcastle Business School, Northumbria University, City Campus East, Newcastle upon Tyne, UK

**Keywords:** Occupational therapy, positive risk-taking, intermediate care, decision making under risk, discharge assessment

## Abstract

**Introduction:**

Positive risk-taking in occupational therapy intermediate care is a requirement, yet little is known about how positive risk-taking barriers influence discharge decisions at different experience levels.

**Method:**

A factorial survey was used to investigate positive risk-taking barriers (Limited Capacity, Risk Averse Family, Blame Culture and No Support). Participants self-categorised their experience level into Novice or Semi-expert or Expert before analysing four vignettes relating to recommending a home discharge for an older adult. Data were analysed using Multiple Regression and One-Way Analysis of Variance.

**Results:**

Seventy-four participants responded to two hundred eighty-one vignettes. The barriers that reduced the likelihood to recommend a home discharge for an older adult were ‘No Support’, Novices (β = −0.315, *p* = 0.002), Semi-experts (β = −0.313, *p* = 0.001) Experts (β = −0.254, *p* = 0.009); ‘Limited Capacity’, Novices (β = −0.305, *p* < 0.003), Semi-experts (β = −0.254, *p* = 0.006) Experts (β = −0.376, *p* = 0.001) and ‘Blame Culture’ Semi-experts (β = −0.240, *p* = 0.010). Novices were found to be less likely to recommend a home discharge by comparison.

**Conclusion:**

The ‘Limited Capacity’, ‘No Support’ and ‘Blame Culture’ barriers had the strongest effect and Novices were less likely to recommend a home discharge overall. These findings could inform future research and pre-registration Occupational Therapy education.

## Introduction

Positive risk-taking is a key risk strategy in occupational therapy intermediate care, especially, when older adults are discharged from hospital to home where resources and support provision may be limited. Positive risk-taking involves mitigating risk whilst also promoting beneficial and appropriate risk taking as part of occupational therapy intervention. Making these types of decisions requires ‘weighing up the potential benefits and harms of exercising one choice of action over another. . .’ (Morgan, 2004: 18). This also means that the repercussions from these decisions may have a favourable or unfavourable outcome or both (Carson, 2008). Making effective positive risk-taking decisions promotes a service user’s progress, enhances safe engagement with occupation, and reduces the potential for missed therapeutic opportunities (Gallagher, 2013). Engaging with risk in this context also empowers service users to challenge the confines of disability (RCOT, 2017). Despite positive risk-taking being endorsed as an appropriate risk strategy in occupational therapy (RCOT, 2017) and intermediate care (NICE, 2017), little is known about how positive risk-taking is employed for older adults with complex needs transitioning from hospital to home. This includes how positive risk-taking barriers might affect decisions of occupational therapy students and occupational therapists at different levels of experience (Newman et al., 2022).

Occupational therapy is a complex intervention in part because it includes engaging with risk and this is a vague area of practice often without ‘hard’ rules and specific procedures (Pentland et al., 2018). Making decisions under risk can include weighing up factors that are extrinsic to the client and may involve reconciling issues of accountability and legality, blame culture, professional risk, organisational resources and moral and ethical dilemmas (Moats and Doble, 2006; Morgan, 2004). These types of factors are likely to detract from making effective risk-related decisions in student and novice occupational therapists above that of experts who have developed robust clinical reasoning and risk management skills.

Experts can use many types of clinical reasoning simultaneously and this can be non-linear, intuitive and involve tacit knowledge (Carrier et al., 2010; Robertson, 2012; Schell and Schell, 2008). Moreover, they can move between intuitive and analytical approaches, and this is often dependent on the task or familiarity with the problem presented (Dhami and Thomson, 2012). Where an analytical approach is required, experts have more confidence in their diagnostic skills (Strong et al., 1995). Using factors as part of a diagnostic process is important to solving problems in a clinical context and assessing risk. Diagnostic reasoning is explained in terms of cues of information which are used as tentative factors to be tested (hypotheses) which can support or disconfirm a course of action. This process involves cue acquisition, followed by hypothesis generation, cue interpretation and hypothesis evaluation in a problem-solving context (Rogers and Holm, 1991). Novices by virtue of having less experience have been shown to be more reliant on analytical and procedural approaches to clinical reasoning (Strong et al., 1995). In the absence of a clear procedural framework, novices may not recognise relevant factors or may be more susceptible to misjudging their importance and or misinterpreting the risk associated to them. In a scoping review, (Newman et al., 2022) found risk management was not the explicit focus of occupational therapy research and this included how positive risk-taking was strategised and how risk was reconciled within occupational therapy intervention.

Risk perception is a subjective process, a precursor to how risk is assessed and has cognitive and emotional dimensions (Paek and Hove, 2017). Risk perception has been shown to be influenced by biased media coverage and misleading personal experiences causing risks to be misjudged (over-estimating or underestimating) with unwarranted confidence (Slovic, 1987; Breakwell, 2007). Occupational therapists are known to make decisions drawing on multiple factors, consciously and unconsciously (Thomas et al., 2019). Potentially there can be an overwhelming amount of information to make decisions and the human brain is constrained by a limited capacity to process information (Newell and Simon, 1972). To maintain a relatively effective means of interpreting the high levels of social information, decision making often involves applying ‘cognitive rules of thumb’, commonly referred to in social psychology as heuristics (Kahneman et al., 1982; Murray and Thomson, 2010). In risk perception, heuristics are employed to help make sense of an uncertain world and whilst helpful in some circumstances, in others they lead to persistent biases (Slovic, 1987; Breakwell, 2007). In relation to a potential risk bias in occupational therapy, risk avoidance and becoming overly cautious to the extent of being risk averse in practice have the capacity to create a harmful situation and inhibit a service user’s progress (RCOT, 2017).

Risk averse perceptions and misjudgements in risk appear to be less influential in experts compared to lay persons with expert risk perceptions being more veridical to the available empirical evidence as well as novices being risk averse by comparison (Rowe and Wright, 2001; Thomson et al., 2004). To a lesser extent this is also true of expert risk perceptions outside of their field of expertise (Breakwell, 2007). Atwal et al. (2012) found that the perception of risk does have an impact on discharge decision-making and that teamwork and collegiality were used by therapists to manage and share risks.

The discharge process in intermediate care is a risk-prone area of practice encompassing risk management to ensure patient safety (during provision) and positive risk-taking (post provision). Davis (2017) found that occupational therapists consider falls risk, living alone, a prolonged stay in hospital and decreased functional or cognitive capacities criteria for a home visit as part of the discharge process. Nygård et al. (2004) assert that pre-discharge home visits are necessary to ensure a patient’s safety and found that occupational therapists were generally in agreement with the client’s responses except when putting themselves at risk. In their literature review, Moats and Doble (2006) identified factors that contribute to risk avoidance in home discharge-related decisions. These factors included conflicting ethical principles of beneficence and autonomy which result in persuasive measures to resolve ethical dilemmas. Additionally, family members sometimes fail to respect risk-taking choices of older relatives in fear of health workers’ condemnation and/or legal reprisals.

Occupational therapists and occupational therapy students introduced to autonomous practice in their fieldwork placements are required to assess the likelihood and potential harm of all risk factors relating to their client prior to recommending a course of action (RCOT, 2017). In consideration that intermediate care occupational therapy is fundamental to ensuring a discharge to home transition is safe and timely, there is a lack of empirical study on the risk management methods employed to facilitate this. Moreover, there is a lack of knowledge on the effect of positive risk-taking barriers at different levels of occupational therapy experience and whether they have the capacity to reduce the likelihood to recommend a discharge to home (Newman et al., 2022).

This study is part of a larger programme of research which has employed a Nominal Group Technique (NGT) of experienced intermediate care occupational therapists to reach consensus on the most common positive risk-taking barriers. In order of the highest ranked items, ‘Limited Capacity’, ‘No Support, ‘Blame Culture’ and ‘Risk Averse Family’ were prominent positive risk-taking barriers. These factors formed the basis of vignettes which were designed to be employed in a factorial survey where their complexity could be manipulated to test their effect strength on novice, semi expert and expert levels of experience.

The aims of this study were to:

- Investigate the effect strength of four positive risk-taking barriers (Limited Capacity, No Support, Risk Averse Family, Blame Culture) on Novice, Semi-expert and Expert occupational therapists in relation to whether they would recommend a home discharge for an older adult.
- Identify which group is more or less likely to recommend a home discharge by comparison.

## Method

This is an online factorial survey study which was approved by the university’s Research Integrity and Ethics Department. All participants provided informed consent for participation, which included the study’s risks and benefits and that their participation was voluntary.

A factorial survey is suitable for studying complex decision making and addresses methodological difficulties in studying how professionals make decisions in real life (Taylor, 2005). True-to-life vignettes are presented to the decision maker who is asked to make a judgement about a familiar scenario (Barter and Renold, 1999; Hughes and Huby, 2004). In a factorial survey, factors, in this case positive risk-taking barriers, are randomised within the vignettes. This gives the factorial survey the robustness of an experimental method and scope for generalisability by using survey methodology to reach larger samples (Ludwick et al., 2004; Taylor, 2005).

The factors used in this survey were informed by a scoping review (Newman et al., 2022) and a consensus study by Nominal Group Technique (NGT). The scoping review investigated the common areas of risk and risk characteristics in intermediate care from an occupational therapy perspective. The NGT convened a panel of experienced intermediate care occupational therapists to reach consensus on the common areas of risk in intermediate care and positive risk-taking barriers. This information was used to construct hypothetical and realistic vignettes, which were subsequently evaluated by the NGT occupational therapists in an advisory capacity to ensure their validity. Additionally, those factors which had reached a high level of agreement in the NGT were considered for this study. This ensured that only the most relevant factors (independent variables) would be included and that potentially these factors would have a strong effect on a decision whether to recommend a home discharge (dependent variable).

A General Data Protection Regulation (GDPR) compliant web-based survey platform was used which randomised the factor levels equally within the vignettes. Ethical approval was obtained prior to recruitment and administration of the survey.

## Sample

Occupational therapy students and occupational therapists were recruited from two university pre-registration programs, the Royal College of Occupational Therapists Specialist Sections – Older People and Trauma and Musculoskeletal Health. Additionally, occupational therapy-related social media was used for recruitment. All participants were asked to categorise themselves into whether they believed they were a Novice or Semi-expert, or Expert in older adult occupational therapy. No other demographic information was sought. Sampling novices and experts for comparison purposes to investigate risk has shown differences in risk perception and risk-related decision making (Rowe and Wright, 2001; Thomson et al., 2004).

As the purpose of this study was to measure the relationship between the factors and the decision made, multiple regression analysis was used and informed the sample size. In relation to this analysis method, a priori power analysis was conducted using G*Power version 3.1.9.7 (Faul et al., 2009) to determine the minimum sample size required to test whether the four independent variables would produce an effect on the dependent variable (likelihood to recommend a home discharge). The required sample size to achieve 80% power for detecting a medium effect, at a significance criterion of α = 0.05, was *n* = 85 participants. The sample size was also informed by the fact that in a factorial survey, the unit of analysis is each vignette and not the participant (Ludwick et al., 2004); as such a minimum of 85 answered vignettes would be required for each group (Novice, Semi-expert and Expert).

Prior to taking the survey, the participants were provided with information advising them of the purpose of the study, the data collection and data storage methods. This information also informed the participants about their right to withdraw, confirmed the survey platform was GDPR compliant and that the study had been granted ethical approval. Additionally, the participants were required to consent to take the survey prior to providing any demographical information or answering the vignettes.

## Data collection

The factorial survey consisted of three sections, an introduction (participant information, demographical information and consent), a background vignette and survey vignettes.

The background vignette was the same for all participants and served to orientate the participants to their hypothetical role. This role was that they were an occupational therapist in a multidisciplinary team conducting hospital to home discharge assessments alongside carers, family and other healthcare workers. Four assessments (vignettes) posed a moderate level of risk and a need for occupational therapy was made explicit; however, further considerations of the four factors (were required) in the context of the changeable variation of each vignette scenario.

All participants were then invited to answer four survey vignettes; these vignettes were arranged into an introduction paragraph followed by the positive risk-taking barriers (factors) written as four bullet points. The four factors were split into two levels (independent variables) with each barrier having an opposite facilitator; this represents a 2×2×2×2 design which produced 16 possible vignette combinations for randomisation. This meant there would be no repetition of a vignette scenario for any of the individual participants answering the survey. Additionally, for each participant, two vignettes related to a male client and two vignettes related to a female client. The vignette introduction and factor levels can be seen in Table 1.

**Table 1.** Vignette text and factor levels.

Vignette and factors	Vignette text and integration of the factor levels	
Survey vignette. The survey randomisation function generated a barrier or facilitator for each factor.	You attend a pre discharge home assessment for Mr/Mrs XXXX, he/she is in his/her 80’s and currently manages his/her medication for two or more medical conditions. He/She has some difficulty performing his/her activities of daily living and has a mild to moderate cognitive impairment. He/She has fallen but not within the last year. Additionally, you consider the following during your risk assessment:	
	Factor 1 (Limited Capacity or Capacity)	
	Factor 2 (Risk Averse Family or Risk Accepting Family)	
	Factor 3 (Blame culture or No Blame Culture)	
	Factor 4 (No Support or Support)	
	Barriers	Facilitators
Factor 1	You are concerned that your recommendations will not be fully understood and carried out as intended	There is a good level of understanding to carry out your recommendations as intended
Factor 2	The client’s family do not want any risks to be taken resulting from discharge ·	The client’s family do understand that risk is something to be managed
Factor 3	You feel if something goes wrong there is a blame culture within the organisations or from colleagues you are coordinating discharge with	There is no blame culture within the organisations or from colleagues you are coordinating discharge with
Factor 4	The family can provide no consistent support after discharge	The client is receiving support from their family

At the end of each vignette, the participant was asked to rate the likelihood of whether they would recommend a home discharge on a scale from 1 (Not Likely) to 7 (Very Likely).

Reliability of the vignettes as the data collection tool was evaluated using a test-retest method on IBM SPSS as part of an intraclass correlation coefficient analysis. Intraclass correlation coefficient (ICC) is a widely used reliability index in test-retest, intrarater and interrater reliability analyses (Koo and Li, 2016).

Three occupational therapy students studying at pre-registration level and five post-registration occupational therapists were recruited for the pilot study using a convenience sampling method. An individualised survey and weblink for each participant were created. The participants were instructed that the survey was to be completed twice, leaving a minimum of 7 days before taking the survey for a second time. The second survey was a duplicate of the first and was sent to each participant after the 7 days had expired. The pilot study participants also completed an evaluation form to comment on the survey presentation, whether the completion time was realistic and whether they felt their risk judgements were tested. The evaluation forms were returned by email.

## Piloting the vignettes and survey instrument

Eight (*n* = 8) occupational therapists completed two surveys leaving 7 days in-between. Both sets of survey results were compared using the ICC method. The ICC in this study was calculated using single ratings, absolute agreement, a two-way mixed effects model with eight participants providing 32 responses for each vignette. A good degree of reliability was found between the same raters when comparing each of their individual responses on these two occasions. The average measure ICC was .858 with 95% confidence interval from 0.729 to 0.928, (F (31/31) = 12.713, *p* < 0.01). Additionally, participant feedback from the five returned evaluation forms confirmed the pilot survey took between 10 and 20 minutes to complete, the vignettes portrayed realistic risk-prone discharge scenarios, the survey was presented clearly and there was sufficient participant information for consent.

## Analysis

Multiple regression analysis was used for Novice, Semi-expert and Expert data sets separately using the ‘Enter’ method on IBM SPSS Statistics v.27. The multiple regression was conducted to determine the effect strength of the barriers to positive risk-taking (i.e. Limited Capacity, Risk Averse Family, Blame Culture and No Support) as independent variables (IV) on the dependant variable (DV), that being, the likelihood to recommend a home discharge. Additionally, a one-way analysis of variance (ANOVA) was performed using IBM SPSS Statistics v.27.

Multiple regression is commonly used for factorial survey analysis, as regression allows the assessment of the relationship between the IVs, and between the IVs and the DV (Hox et al., 1991; Ludwick et al., 2004; Taylor, 2005). Dummy coding was used to convert the IVs from nominal variables to scale variables using 0 for a facilitator and 1 for a barrier.

Multiple regression analysis assumes that the data has certain characteristics and these assumptions, when met, support the accuracy and reliability of the results. Prior to conducting multiple regression, the researchers confirmed there was a linear relationship between the IVs and the DV and that there were no independent variables that were highly correlated (multicollinearity) that could potentially lead to the misinterpretation of the effect strength of any of the IVs. Additionally, it was confirmed that each observation in the data sets was independent and that the residuals from the data had constant variance (homoscedastic) at every point in the regression model. This includes that the residuals were normally distributed and there were no influential responses that could bias the analysis (Cohen, 2003; Osborne and Waters, 2002).

A One-way analysis of variance (ANOVA) was performed using the mean scores of the DVs for each group to determine whether there was a statistically significant difference between Novices, Semi-experts and Experts’ propensity to recommend a discharge. A Bonferroni correction was also used to adjust the probability (*p*-values) because of the increased risk of a Type 1 error when calculating multiple statistical tests (Armstrong, 2014).

## Results

### Factorial survey analysis

A total of 109 participant responses were recorded with 74 participants answering one or more of the vignettes. Twenty-two (*n* = 22) 26% participants indicated they were occupational therapy students and sixty (*n* = 62) 74% indicated they were qualified occupational therapists. Participants self-categorised their experience level into Novice, Semi-expert or Expert in older adult occupational therapy. Eight (*n* = 8) Novices indicated they were post-registered occupational therapists, two Semi-experts indicated they were occupational therapy students (pre-registration), all Experts were occupational therapists (post registration). Twenty-three (*n* = 23) Novices answered 87 vignettes, 26 (*n* = 26) Semi-experts answered 99 vignettes and 25 (*n* = 25) Experts answered 95 vignettes; 281 vignettes in total were answered. Thirty-two percent (32%) of participants dropped out prior to answering any of the vignettes.

The positive risk-taking barriers (independent variables) were entered together as a model to investigate their combined and individual effect. All positive risk-taking barriers produced a negative effect overall and reduced the likelihood of recommending a discharge to home for an older adult. Additionally, their collective effect as a model was statistically significant in all groups.

In the Novice group, this model explained 21.9% variance in the likelihood to recommend a discharge to home (*R*^2^ = 0.219, *F* (4,82) = 5.734, *p* < 0.001). Out of the four IVs in this model, two were found to be statistically significant, No Support (β = −0.315, *p* = 0.002) and Limited Capacity (β = −0.305, *p* < 0.003). A negative coefficient was observed for the IVs confirming their presence reduced the likelihood to discharge. Holding all IVs constant, those that were statistically significant in the model were observed to reduce the DV by at least 1 point on the 7 point scale, for No Support (*B* = −1.063, CI −1.734,−0.392) and for Limited Capacity (*B* = −1.031, CI −1.713,−0.349). The IVs in the Novice multiple regression model were ranked in relation to their statistical significance; see Table 2.

**Table 2.** Novice group, multiple regression analysis and factor ranking.

Ranking	Factor	β	*p*-Value*
1	No support	−0.315	0.002
2	Limited capacity	−0.305	0.003
3	Risk averse family	−0.087	0.399
4	Blame culture	−0.069	0.487

* Two tailed.

In the Semi-expert group, this model explained 23.5% variance in the likelihood to recommend a discharge to home (*R*^2^ = 0.235, *F* (4,94) = 7.238, *p* < 0.001). The factors, No Support (β = −0.313, *p* = 0.001), Limited Capacity (β = −0.254, *p* = 0.006) and Blame Culture (β = −0.240, *p* = 0.010) were found to be statistically significant. These factors produced a negative coefficient on the DV. A decrease in the DV by over 1 in the 7-point scale was found for No Support (*B* = −1.087, CI −1.723, −0.450), this effect was less for the Limited Capacity (*B* = −0.877, CI −1.500, −0.254) and the Blame Culture (B = −0.831, CI −1.456, −0.207) independent variables. The IVs in the Semi-expert multiple regression model were ranked in relation to their statistical significance; see Table 3.

**Table 3.** Semi-expert group, multiple regression analysis and factor ranking.

Ranking	Factor	β	*p*-Value*
1	No support	−0.313	0.001
2	Limited capacity	−0.254	0.006
3	Blame culture	−0.240	0.010
4	Risk averse family	−0.076	0.413

* Two tailed.

In the Expert group, this model explained 21.8% variance in the likelihood to recommend a discharge to home (*R*^2^ = 0.218, *F* (4,90) = 6.254, *p* < 0.001). The factors, Limited Capacity (β = −0.376, *p* = 0.001) and No Support (β = −0.254, *p* = 0.009) were found to be statistically significant. These factors produced a negative coefficient on the DV. When the Limited Capacity (B = −1.173, CI −1.768, −0.579) factor was present, this caused the DV to decrease by over 1 in the 7-point scale; this effect was not as strong in the No Support independent variable (B = −0.799, CI −1.394, −0.205). The IVs in the Expert multiple regression model were ranked in relation to their statistical significance; see Table 4.

**Table 4.** Expert group, multiple regression analysis and factor ranking.

Ranking	Factor	β	*p*-Value*
1	Limited capacity	−0.376	0.001
2	No support	−0.254	0.009
3	Blame culture	−0.169	0.078
4	Risk averse family	0.029	0.758

* Two tailed.

### One-way ANOVA

The One-way ANOVA found the Novice group were neutral being neither unlikely or likely to recommend a home discharge, with a mean of 4.28. The Semi-expert and Expert groups were likely to recommend a home discharge with a mean of 5.03 and 5.45, respectively; see Table 5.

**Table 5.** Dependent variable mean differences by experience level.

Group/Experience level	*N*	Mean	Std. Deviation	Std. Error
Novice	87	4.2874	1.69752	0.18199
Semi Expert	99	5.0303	1.73473	0.17435
Expert	95	5.4526	1.56945	0.16102
Total	281	4.9431	1.72905	0.10315

A One-way ANOVA revealed that there was a statistically significant difference in the mean to recommend a home discharge (DV) between at least two groups (*F*(2,278) = 11.279, *p* = 0.001). Multiple comparison analysis of the One-way ANOVA results confirmed statistical significance between the means of Novice and Semi-experts (*p* = 0.008 CI −1.33, −0.15) and Novice and Experts (*p* = 0.001 CI −1.76, −0.56) but not between Semi-experts and Experts.

## Discussion

This factorial survey has afforded a means to investigate a number of positive risk-taking barriers in relation to discharge decisions made by occupational therapists; these would be difficult to investigate in occupational therapy practice. It has identified the effect strength of the positive risk-taking barriers (Limited Capacity, Risk Averse Family, Blame Culture and No Support) on the likelihood to recommend a home discharge in relation to three different occupational therapy experience levels Novice, Semi-expert and Expert. It has also found that these experience levels have different propensities to discharge in relation to these barriers. The Novice group were least likely to recommend a home discharge in relation to the positive risk-taking barriers in comparison to the Semi-expert and Expert groups, indicated by their neutrality to recommend a home discharge.

The effect strength of the ‘No Support’ and ‘Limited Capacity’ factors on all groups was statistically significant. The ‘Blame Culture’ factor was statistically significant on the decisions of Semi-experts, whereas the decisions of Novices and Experts in relation to the blame culture positive risk-taking barrier were not affected at a statistically significant level. The ‘Risk Averse Family’ factor related to the client’s family not supporting risk taking and this was found not to have any significant effect on any of the groups’ decisions whether to recommend a home discharge.

In both the Novice and Semi-Expert groups, ‘No Support’ was the factor which was found to have the most effect strength, and for Experts this was second to ‘Limited capacity’. Previous literature also highlights the importance of lack of support in this area. Zurlo and Zuliani (2018) assert that discharge planning starts with a patient’s admission, finishing with the patient being discharged to a setting able to support them in the best way possible. Moats and Doble (2006) postulate, individuals are discharged from acute care hospital settings after brief stays, and in some cases, before adequate supports have been put in place or steps taken to reduce risks. In their systematic review on the risk factors associated with hospital readmission, García-Pérez et al. (2011) found that poor family support was a risk factor for three or more hospital readmissions over a period of 6 months. This is consistent with the finding from this study that ‘No Support’ was a significant factor in reducing the likelihood of a recommendation to discharge across all occupational therapy experience levels.

In the Expert group, ‘Limited Capacity’ was found to have the strongest effect strength on a decision to discharge to home and was second to ‘No Support’ in the Novice and Semi-expert groups. Previous research has shown that older adults leaving hospital can be highly variable to rehabilitation (McIntyre and Atwal, 2005) and those with ‘limited capacity’ through diseases like dementia can exhibit many unsafe behaviours which are hard to predict, be resistant to changing such behaviours or have difficulty complying with interventions targeted to keep them safe (Lach and Chang, 2007).

The implications of this are complex. Having decision-making capacity is a prerequisite for understanding the potential benefits and harms in occupational therapy interventions which employ positive risk-taking strategies. In the context of discharge decisions, Moats (2007) found occupational therapists described the need to strike a balance between maintaining safety and respecting autonomy. However, ‘where clients were cognitively impaired but not officially ‘incompetent’ (a situation which occurs frequently), the processes described by therapists were more complex and ill-defined’ (Moats, 2007: 96). Morgan and Andrews (2016) assert, mental capacity is specific, not a generalized attribution to a person; so, a focus on a person’s understanding of the choices and consequences should be related to a specific decision and ‘enabling’ people should focus on capability rather than disability. This is an important tenet of the Mental Capacity Act 2005 (Legislation.gov.uk, 2005) but its implementation in relation to positive risk-taking has suffered from a lack of awareness of its central role (Morgan and Andrews, 2016). Moreover, when autonomy promotion is the prevailing consideration and decisional control rests with the client, this can become challenging when risks are high, as such, these situations may result in ethical dilemmas (Moats and Doble, 2006). Such dilemmas occuring when promoting a client’s autonomy may also mean that safety cannot be assured, optimised or completely reconciled within the complexity of a client’s occupational routine. Unpredictable and unsafe client behaviours makes assessing risk particularly challenging and with the potential to cause ethical dilemmas is likely to explain why the ‘Limited Capacity’ factor had a strong effect in all groups and the most effect on Experts.

When making a decision on discharge to home, ‘Blame culture’ was found to be a statistically significant factor in the Semi-expert group only. This may indicate that blame culture does not impact early career occupational therapists or student occupational therapists who either have little working experience or are perhaps more protected from such conditions whilst in practice placement and preceptorship phases of their career. The stronger effect strength of ‘Blame culture’ within the Semi-expert group may signify that this group feels more susceptible to being blamed for errors, perhaps as they transition from novice to more autonomous practice but before they attain higher levels of expertise.

The implications of this are important for organisations employing occupational therapists. In consulting to produce risk guidance, the Department of Health (DOH) found there is a genuine fear of adverse consequences when empowering people to take risks where, if things go wrong, blame was likely to be directed at organisations or individuals (DOH, 2007). Changing a blame culture to a learning culture is imperative to protect staff if ‘true’ mistakes are made, to share information and to learn (as an organisation) from practice errors or near misses (DOH, 2010). Working within a culture of blame has been found to be associated with avoiding reporting errors owing to the fear of retribution (Okpala, 2020). Fear can be a strong ‘affect heuristic’ which has the capacity to alter cognitive processes, potentially biasing learning, memory and/or decision making. Furthermore, Morgan (2004: 19) states ‘. . .positive risk-taking becomes undermined, as the fears associated with a blame culture are more likely to permeate people’s thinking and threaten the implementation of creative ideas’.

This study has identified that the likelihood to recommend a risk-prone home discharge increases with experience and that ‘Limited Capacity’, ‘No Support’ and ‘Blame Culture’ are important decision-influencing factors. The factor with the most effect strength on Experts was ‘Limited Capacity’. A person’s capacity can fluctuate and be unpredictable and this is difficult to objectify in a risk assessment to employ positive risk-taking. Considering this finding, training early career occupational therapists would be advantageous in light of such complexity and the challenges associated with older adult discharge. Moreover, further research on positive risk-taking with older adults during discharge where there are different levels of support and/or a client’s cognitive capacity would be beneficial to occupational therapy education and practice. Additionally, organisations and senior occupational therapists should be aware of the particular impact of blame culture on more junior occupational therapists as they transition to higher levels of expertise and should ensure that appropriate support systems are in place.

To the authors’ knowledge, this is the first study of its kind to investigate the effect strength of positive risk-taking barriers in the context of an older adult discharge. It has used an innovative method (factorial survey) to embed (implicitly) and randomise the factor levels into the vignettes as part of an experimental and systematic approach. This has facilitated the elucidation of the factors which carry the most weight in relation to the three groups of occupational therapy experience (Novice, Semi-expert and Expert); however, a limitation of the study is that participants self-categorised their experience level. This study benefits from the experience of a multi-professional research team. Despite 281 vignettes being answered in this study, there was a moderate non-completion rate of 32% where thirty-five participants did not answer one vignette. This study did not seek to emphasise the combined effect of the factors contained within the multiple regression model as a predictive model for risk. However, each of the models’ proportion of variance in the dependent variable (likelihood to recommend a discharge to home) for Novices, Semi-experts and Experts were low as indicated by the R^2^ values reported.

## Conclusion

In this study, as level of experience increased so did the likelihood to recommend a home discharge. The positive risk-taking barriers, ‘No Support’, ‘Limited Capacity’ and ‘Blame culture’ had the strongest effect overall, with the barriers ‘No Support’ and ‘Limited Capacity’ having a statistically significant effect on the likelihood to recommend a home discharge for all experience levels. The positive risk-taking barrier, ‘Blame Culture’ had a statistically significant effecton the Semi-experts but not on the Novices or Experts.

In the Expert group ‘Limited Capacity’ had the strongest effect, and this factor is multifactorial and challenging to reconcile with balancing autonomy and safety considerations in positive risk-taking. In consideration of its effect on experts emphasises the importance for further research. Moreover, research should further investigate the positive-risk taking barriers ‘No Support’ and ‘Blame Culture’ to develop a greater understanding of the impact of these factors on discharging older adults with complex needs. This research should also be directed at understanding the experiences of occupational therapists in relation to these factors.

This study has identified the positive risk-taking barriers which have the strongest effect on different levels of experience. These findings could be used as a targeted approach to training occupational therapists at pre-registration level, inform intermediate care practice and benefit occupational therapists who are new to discharge risk assessment and positive risk-taking. Additionally, this study found that blame culture had statistically significant effects on occupational therapists who have not yet obtained expertise in older adult occupational therapy, although, student therapists and those in the preceptorship phase of their career were not affected at the same level. Organisations and senior occupational therapists should be aware that such a factor can impact the positive risk-taking decisions of lesser experienced therapists and ensure support systems are in place.
